# A Long Short-Term Memory-Based Approach for Detecting Turns and Generating Road Intersections from Vehicle Trajectories

**DOI:** 10.3390/s22186997

**Published:** 2022-09-15

**Authors:** Zijian Wan, Lianying Li, Huafei Yu, Min Yang

**Affiliations:** 1 School of Resource and Environmental Sciences, Wuhan University, Wuhan 430079, China; 2 Department of Geography, University of California, Santa Barbara, CA 93106, USA

**Keywords:** road intersection, turn detection, vehicle trajectories, long short-term memory

## Abstract

Owing to the widespread use of GPS-enabled devices, sensing road information from vehicle trajectories is becoming an attractive method for road map construction and update. Although the detection of intersections is critical for generating road networks, it is still a challenging task. Traditional approaches detect intersections by identifying turning points based on the heading changes. As the intersections vary greatly in pattern and size, the appropriate threshold for heading change varies from area to area, which leads to the difficulty of accurate detection. To overcome this shortcoming, we propose a deep learning-based approach to detect turns and generate intersections. First, we convert each trajectory into a feature sequence that stores multiple motion attributes of the vehicle along the trajectory. Next, a supervised method uses these feature sequences and labeled trajectories to train a long short-term memory (LSTM) model that detects turning trajectory segments (TTSs), each of which indicates a turn occurring at an intersection. Finally, the detected TTSs are clustered to obtain the intersection coverages and internal structures. The proposed approach was tested using vehicle trajectories collected in Wuhan, China. The intersection detection precision and recall were 94.0% and 91.9% in a central urban region and 94.1% and 86.7% in a semi-urban region, respectively, which were significantly higher than those of the previously established local G* statistic-based approaches. In addition to the applications for road map development, the newly developed approach may have broad implications for the analysis of spatiotemporal trajectory data.

## 1. Introduction

Accurate and up-to-date road maps are critical for location-based services, such as vehicle navigation and geo-enabled social networks. Because road entities change continuously over time, especially in the urban areas of developing countries, existing road maps need to be updated regularly to remain consistent with the real world. However, national mapping agencies usually update road maps by surveying with specialized devices or by digitizing road features from high-resolution satellite imagery. These methods require extensive time and labor, making it difficult to maintain up-to-date maps. In recent years, the widespread use of GPS-enabled devices has driven an explosion of trajectory data from road users, such as vehicle drivers. This new type of geospatial resource contains not only geometrical and topological information for underlying road networks but also semantic information, such as traffic rules and patterns. Moreover, short-term changes in moving paths and rules can be sensed from the continuous tracking data, enabling them to be updated in real-time. For these reasons, extracting road information from trajectory data is becoming an attractive method for road map production and updating [1,2].

A number of approaches have been developed to generate road networks from trajectories, including approaches based on spatial clustering [2,3,4], incremental track integration [5,6], and intersection linking [7,8,9,10]. Most of these approaches focus on the extraction of road centerlines, while intersections, defined as areas where two or more roads either meet or cross, are simply recognized as graph nodes where centerlines are connected. In the representation of a road network, intersections can be simple crossroads or various complicated structures, such as roundabouts. Obtaining well-structured intersections is not only essential for building the topology of road networks, but it is also beneficial for obtaining a geometric representation of the roadways. Particularly for urban areas, automatic intersection detection and delineation approaches are highly necessary for various applications, such as traffic management [11], analyses of transportation routes [12], and analyses of urban sprawl [13]. Therefore, intersections must be adequately considered in the generation of road networks.

However, obtaining accurate and well-structured intersections from raw trajectories continues to be a challenging task. From the perspective of traffic engineering, an intersection is designed to allow vehicles to change from one road to another. This means that complex geometrical structures and traffic rules may exist within a small area of an intersection. In one of the earliest attempts to detect intersections, a shape descriptor was designed to represent the heading changes of tracking points around a given location and trained a classifier to discriminate the intersections from non-intersections [7]. Turning points were detected in literature [8] by examining changes in the speed and heading of vehicles. The turning points were then grouped to generate intersections by hierarchical clustering. A spatial analysis was performed of the conflict points among the trajectories that intersect with a large angle to compute the layouts of intersections [14]. To enable the effective detection of turns, literature [15] proposed a distance-weighted average heading filter to eliminate the serious motor-vehicle trajectory fluctuations, and literature [16] employed the Douglas–Peucker compression algorithm to remove noise. Intersections were detected in the literature by [17] using a hierarchical feature extraction strategy. The turning change point pairs were extracted from trajectories by setting the heading and time interval thresholds and were then clustered to obtain the coverage of the intersections. Literature [18] identified candidate intersection points by applying a hotspot analysis of the local G statistic to the turning angles of tracking points. A large positive G* value represents a hotspot cluster in which the points with large angles are closer together. Subsequently, an adaptive clustering algorithm grouped the candidate points to obtain the intersections. In this approach, the detection result is closely related to the threshold G* value. A novel method for constructing a lane-level motorway network was presented using low-precision GPS data [19]. In this study, an intersection was defined where two bundles of trajectories merge or diverge. Based on this definition, the QuickBundles clustering method was used to obtain the trajectory bundles [20]. Then, road intersections were detected though analyzing the intersections of trajectory paths belonging to different bundles. Literature [21] uses Mask-RCNN to extract macroscopic information on road intersections. In addition to the coverage of intersections, this approach attempts to classify the detected intersections into different patterns.

In general, an essential step prior to intersection generation is the recognition of turns (i.e., the curved parts of vehicle trajectories). Turning points are commonly defined as heading changes exceeding a predefined threshold. Then, spatial analysis is applied to cluster the turning points into intersection units and compute each intersection’s properties. However, these approaches face usability problems. Owing to the diversity of intersection patterns and sizes, it is difficult to determine an appropriate turning angle threshold [16,22,23]. A low or high threshold inevitably leads to incorrect turn detection. Furthermore, quality issues in raw trajectory data, such as fluctuating positional precision and inconsistent sampling rates, increase the difficulty of characterizing the turns occurring at intersections. Other indicators, such as the speed change in the vehicle and the curvature of the segment formed by connecting consecutive tracking points, should be integrated with the change in direction to improve the performance of turn detection.

To address the aforementioned issues, this study introduces a deep learning technique to detect the turns at intersections from raw trajectories, aiming to alleviate the difficulty of parameter setting in turn detection and to develop a more robust method that can benefit from the movement pattern information hidden in each trajectory. Through continuous training, deep neural networks can capture high-level features and identify hidden patterns [24]. Among various neural networks, the long short-term memory (LSTM) neural network is an effective model for handling time-series data. As an improved version of the traditional recurrent neural network, the LSTM neural network has the ability to capture long-term dependencies in sequencing data [25,26]. Previous studies have shown the successful application of LSTM in the fields of natural language processing [27], machine translation [28], and speech recognition [29]. Recent studies have also applied LSTM-based models for trajectory analysis tasks, including trajectory clustering [30], transportation mode classification [31], and location prediction [32,33]. In this study, we explore the potential of LSTM to detect intersections from trajectories. Specifically, we convert each trajectory into a feature sequence that stores the motion attributes of the vehicle along the trajectory. Subsequently, an LSTM-based model is trained to identify the turning trajectory segments (TTSs), each of which represents a turn occurring at an intersection, by capturing a deep representation of the input feature sequence. Finally, the detected TTSs are clustered to generate the coverage and structure of the intersections.

The remainder of this paper is organized as follows. The proposed approach is explained in Section 2. Section 3 presents the experimental datasets, intersection detection results, and detailed analyses. A general discussion is presented in Section 4, and key areas for future work are described in Section 5.

## 2. Methods

**Figure 1:**
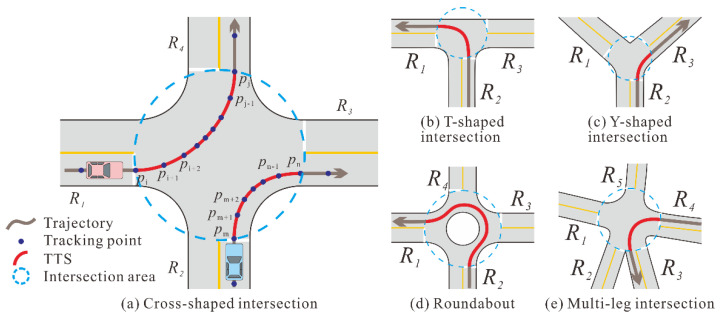
Samples of turning trajectory segment (TTS) at different types of intersections.

We represent the structure of an intersection as a network graph with a simple circular area. When a vehicle turns within an intersection, the TTS starts from the first tracking point entering the boundary circle and ends with the last point leaving it. As illustrated in Figure 1a, the trajectory segment from $p_{i}$ to $p_{j}$, denoted as $TS\left(p_{i},\;p_{j}\right)$, and the trajectory segment from $p_{m}$ to $p_{n}$, denoted as $TS\left(p_{m},\;p_{n}\right)$, are two TTSs that record the left and right turns at a cross-shaped intersection, respectively. Moreover, $p_{i}$ and $p_{m}$ indicate the entry points, while $p_{j}$ and $p_{n}$ are located near the exits of the intersection. Figure 1 shows the TTS samples for the various intersection types.

**Figure 2:**
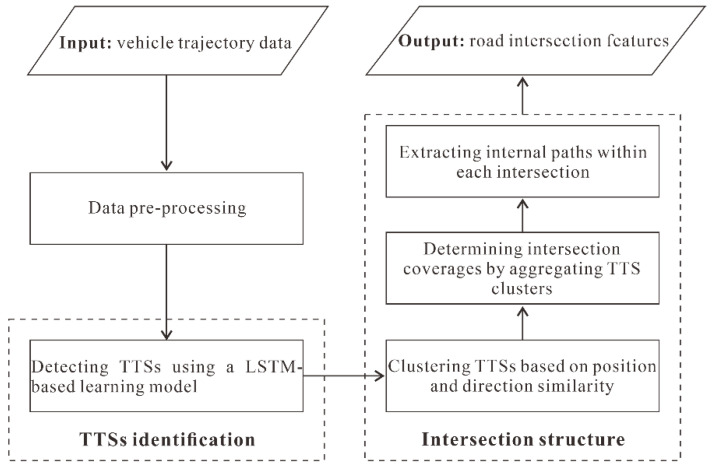
Framework of the proposed approach for detecting intersections from trajectories.

The overall framework of the proposed approach consists of two parts, as illustrated in Figure 2. Specifically, the two components are:

(1) TTS detection: this component identifies the TTSs contained in each trajectory using an LSTM-based model that integrates the various motion attributes implied in the tracking points.
(2) Intersection generation: this component calculates the TTS clusters based on the similarity of the position and direction measures and then determines the coverage of the intersections by aggregating the TTS clusters and extracting the internal paths of each intersection.

### 2.1. Data Pre-Processing

Owing to various uncertain factors, the raw trajectories must be cleaned by reducing the outliers and random errors. Initially, the tracking points for each vehicle are ordered chronologically as a trajectory. When the time interval between two consecutive points exceeds a predefined threshold, intTh, the trajectory is split into two sub-trajectories. Moreover, the tracking points with speeds exceeding the maximum valid speed threshold maxV are labeled invalid. After removing the invalid tracking points, the trajectories whose lengths are smaller than the threshold minLen are also discarded.

An interpolation operation with a distance threshold, dl, is performed for each trajectory to ensure that the distances between the pairs of adjacent points are close. Then, the Savitzky–Golay filter is applied to reduce random errors [34]. As a widely used approach for smoothing a time series, the Savitzky–Golay filter can reduce random positional errors in the input trajectories without causing significant distortion of their original shapes and motion features [35]. It replaces each tracking point with a new point obtained from a polynomial fit to data within a window centered at the subject point. Note that the parameter settings during the pre-processing are closely related to the characteristics of the trajectory data.

### 2.2. Detecting TTSs Using an LSTM-Based Model

**Figure 3:**
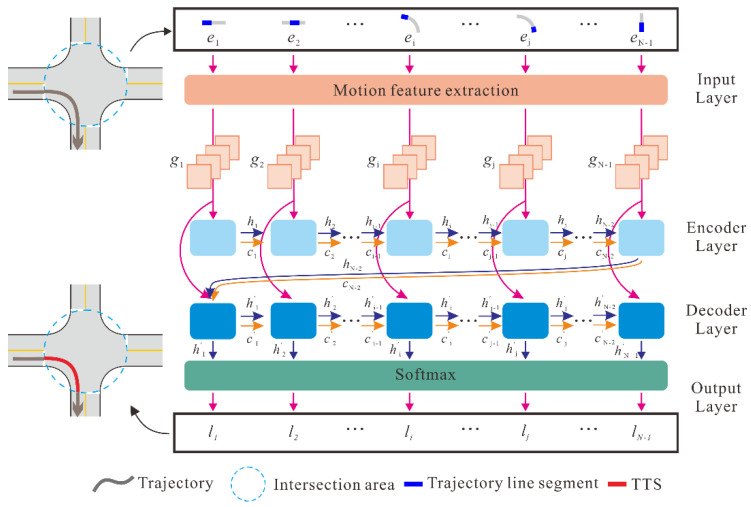
Architecture of the LSTM-based sequence-to-sequence model for TTS detection.

Let T denote a trajectory that contains a sequence of tracking points $p_{1},\;p_{2},\cdots ,\;p_{N}$ with timestamps $\;t_{1},\;t_{2},\cdots ,\;t_{N}\;\left(t_{1}<t_{2}<\cdots <t_{N}\right)$ and $e_{i}\left(i=1,\;2,\cdots ,N-1\right)$ denotes the line segment between the two consecutive tracking points $p_{i}$ and $p_{i+1}$. Detecting TTSs contained in T can be viewed as a classification task, which aims to determine whether each line segment belongs to a TTS or not. For this task, an LSTM-based sequence-to-sequence model is proposed to learn and predict the class of each line segment in a trajectory. Figure 3 presents the architecture of the proposed model, including the input layer, encoder and decoder layers, and output layer.

#### 2.2.1. Input Layer

The input layer converts the representation of *T* into a sequence of vectors {*g*_1_, *g*_2_, …, *g_N_*_−1_}, where *g_i_* (i=1, 2,⋯,N−1) stores the attributes that describe the motion characteristics of the vehicle at line segment $e_{i}$. We traverse a sliding window with constant size s, defined by the number of line segments inside the window, along each trajectory to obtain its motion attribute sequence. When the sliding window is centered at line segment $e_{i}$, four motion attributes, including tortuosity, turning angle, speed and acceleration, are computed according to the tracking points inside the window.

Distinct from traveling on roadways, a vehicle changes its heading substantially when turning at an intersection. Accordingly, we introduce the tortuosity and turning angle as the first two motion attributes. Let $p_{u},\;p_{u+1},\ldots ,\;p_{v}$ denote the tracking points inside the sliding window. The tortuosity and turning angle are computed using Equations (1) and (2), respectively.

(1) $$T\left(e_{i}\right)=\frac{dis\left(p_{u},\;p_{v}\right)}{\sum_{k=u}^{v-1}dis\left(p_{k},p_{k+1}\right)}$$

(2) $$A\left(e_{i}\right)=\{\begin{array}{ll} \theta_{v}-\theta_{u}, & \theta_{v-1}\geq \theta_{u}\;\\ \theta_{v}-\theta_{u}+360,\; & \theta_{v-1}<\theta_{u} \end{array}\;$$

where *dis*($p_{a},p_{b}$) is a function that returns the Euclidean distance from $p_{a}$ to $p_{b}$ and $\theta_{u}$ and $\theta_{v}$ represent the moving headings of tracking points $p_{u}$ and $\;p_{v}$, respectively. The heading of point $p_{k}$ is defined as the clockwise angle between due north and the vector $\vec{p_{k}p_{k+1}}$.

Vehicles usually change their speed at intersections, which provides another indicator to detect turning behavior. In this study, speed and acceleration are adopted as the other two motion attributes and are measured using Equations (3) and (4), respectively.

(3) $$V\left(e_{i}\right)=\frac{\sum_{k=u}^{v-1}dis\left(p_{k},\;p_{k+1}\right)}{t_{v}-t_{u}}\;\;\;$$

(4) $$AC\left(e_{i}\right)=\frac{V\left(e_{i+1}\right)-V\left(e_{i}\right)}{t_{i+1}-t_{i}}\;\;$$

After calculating the four motion attributes of each line segment, a motion attribute sequence with a four-channel structure is constructed for the input trajectory. Note that the sequence length varies among the input trajectories owing to variation in the number of tracking points. To ensure that each instance input to the model has the same size, which is critical for uniformly applying the model weights and biases to the entire batch, a fixed-length processing approach is implemented. Using *MaxLen* (denoting the constant length of a sequence within a batch) as a threshold, longer sequences are sub-divided, and zero values are appended to the end of the shorter sequences.

#### 2.2.2. Encoder and Decoder Layers

**Figure 4:**
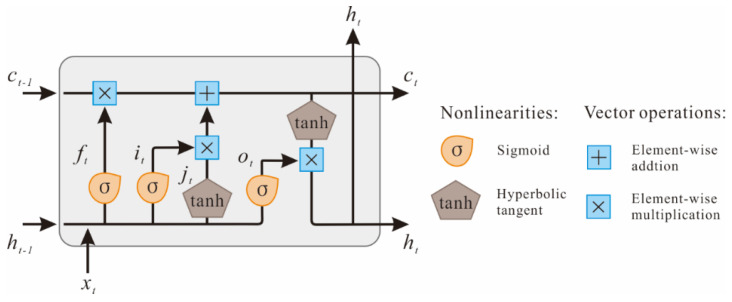
Schematic diagram of an LSTM cell, as proposed in the literature [25].

As shown in Figure 3, the encoder and decoder layers employ two LSTM neural networks. As a recurrent neural network variant, the LSTM effectively overcomes the vanishing and exploding gradient problem by introducing a trainable forget gate [25]. Figure 4 illustrates the structure of an LSTM unit, where $x_{t}$ and $h_{t}$ are the input and output of the LSTM unit at time t, respectively, and $c_{t}$ denotes the cell state of the LSTM unit. In addition, $f_{t}$ denotes the vectors of the forget gate, $i_{t}$ and $j_{t}$ denote the vectors of the input gate, and $o_{t}$ denotes the vectors of the output gate. The LSTM unit works based on the following mechanism.

(5) $$f_{t}=\sigma \left(W_{fx}x_{t}+W_{fh}h_{t-1}+b_{f}\right)\;\;$$

(6) $$i_{t}=\sigma \left(W_{ix}x_{t}+W_{ih}h_{t-1}+b_{i}\right)\;\;$$

(7) $$j_{t}=\tan h\left(W_{j}\cdot \left[h_{t-1},x_{t}\right]+b_{j}\right)\;\;$$

(8) $$c_{t}=f_{t}⨀c_{t-1}+i_{t}⨀j_{t}\;$$

(9) $$o_{t}=\sigma \left(W_{ox}x_{t}+W_{oh}h_{t-1}+b_{o}\right)\;$$

(10) $$h_{t}=o_{t}⨀\tanh\left(c_{t}\right)\;$$

where ⨀ represents the element-wise product, W and b denote the weight matrices and bias vectors, which are adjusted through training. The activation functions sigmoid σ() and hyperbolic tangent tanh() are used for nonlinear scaling.

The encoder sequentially processes the motion attribute sequence {*g*_1_, *g*_2_, …, *g_N_*_−1_}. Once a new vector *g_i_* (i=1, 2, ⋯, N−1) is added to the encoder, the hidden state $h_{i}$ and the cell state $c_{i}$ of the current LSTM unit are calculated based on the input *g_i_*, hidden states $h_{i-1}$, and the cell state $c_{i-1}$ of the previous LSTM unit. After the last vector, *g_N_*_−1_, is processed, the encoder summarizes the entire input sequence into the final states $h_{N-1}$ and $c_{N-1}$. Then, using $h_{N-1}$ and $c_{N-1}$ as the initial states, the decoder recursively generates the output sequence $\left\{{h\prime }_{1},{h\prime }_{2},\cdots ,{h\prime }_{N-1}\right\}$. The output vector ${h\prime }_{i}\;\left(i=1,\;2,\;\cdots ,\;N-1\right)$ for the *i*th decoder LSTM unit is derived by combing vector *g_i_* and the states ${h\prime }_{i-1}$ and ${c\prime }_{i-1}$ that were obtained from the previous decoder LSTM unit.

#### 2.2.3. Output Layer and Training Process

The output layer generates the classification decision for each line segment of the input trajectory *T*. By applying the *Softmax* function to the output vector, ${h\prime }_{i}\;\left(i=1,\;2,\cdots ,\;N-1\right)$, the predicted probability vector $l_{i}=\left(l_{i}^{0},l_{i}^{1}\right)$ is derived for each line segment $e_{i}$, where $l_{i}^{0}$ represents the probability of $e_{i}$ not being a part of a TTS and $l_{i}^{1}$ represents the probability of $e_{i}$ being a part of a TTS. $l_{i}^{0}$ and $l_{i}^{1}$ are both in the interval of (0, 1). If $l_{i}^{1}>l_{i}^{0}$, $e_{i}$ is predicted as a part of a TTS; otherwise, $e_{i}$ is predicted as a part of a non-TTS. The contained TTSs can then be obtained by connecting the consecutive line segments with positive predictions.

The proposed model was trained using a supervised method. The goal of the training process is to learn the optimal parameters that minimize the loss function. In this study, the loss function was calculated using categorical cross-entropy.

### 2.3. Generating Intersection Structures from TTSs

At this stage, the intersections are generated by estimating the spatial distribution of the detected TTSs. First, the detected TTSs are clustered based on the similarity of the position and direction measures. Then, the coverages of the intersections are determined by aggregating the TTS clusters. Finally, the internal road paths for each intersection are constructed.

#### 2.3.1. Clustering TTSs Based on Position and Direction Similarity

This step aims to obtain clusters of TTSs, with each cluster indicating a turning path. Based on previous studies [17], the similarity between two TTSs was measured by estimating the differences in position and direction at their critical points. Suppose $TS\left(p_{a},p_{a+m}\right)$ and $TS\left(p_{b},p_{b+n}\right)$ are the two detected TTSs, and $p_{a+k}$ and $p_{b+l}$ are the middle points of these two TTSs. The position and direction differences between $TS\left(p_{a},p_{a+m}\right)$ and $TS\left(p_{b},p_{b+n}\right)$ are calculated using equations (11) and (12), respectively.

(11) $$\Delta D=\frac{dis\left(p_{a},p_{b}\right)+dis\left(p_{a+k},p_{b+l}\right)+dis\left(p_{a+m},p_{b+n}\right)}{3d}\;$$

(12) $$\Delta A=1-\cos\left(\frac{\left|\theta \left(\vec{p_{a}p_{a+1}},\;\vec{p_{a+m-1}p_{a+m}}\right)-\theta \left(\vec{p_{b}p_{b+1}},\;\vec{p_{b+n-1}p_{b+n}}\right)\right|}{2}\right)\;$$

where $\theta \left(\vec{p_{a}p_{a+1}},\;\vec{p_{a+m-1}p_{a+m}}\right)$ is a function that returns the angle between the vectors of $\vec{p_{a}p_{a+1}}$ and $\vec{p_{a+m-1}p_{a+m}}$, and a constant *d* is utilized to normalize the distance measure during the similarity computation. The overall similarity between the two given TTSs is computed as follows:

(13) $$Sim\left(TS\left(p_{a},p_{a+l}\right),TS\left(p_{b},p_{b+m}\right)\right)=w_{D}e^{-\Delta D}+w_{A}e^{-\Delta A}$$

where $w_{D}$ and $w_{A}$ are the weights related to the distance and direction measures, respectively.

**Figure 5:**
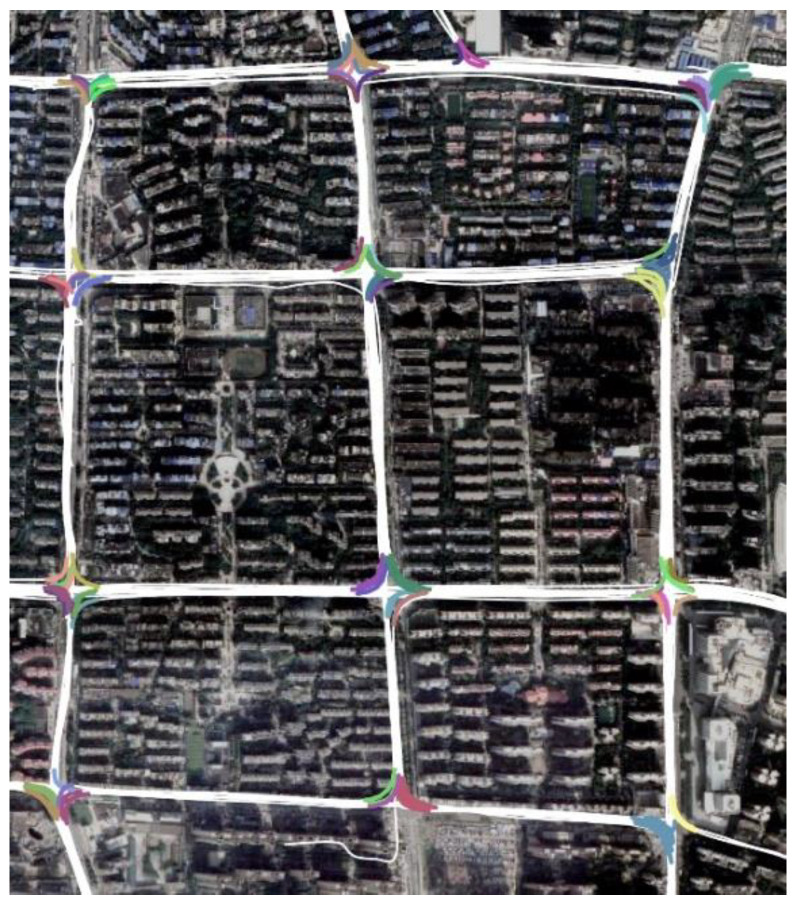
Example of TTS clustering results. Adjacent TTSs marked with the same color belong to one cluster.

Based on the aforementioned similarity model, a seed-based approach was implemented to cluster the detected TTSs. Let $UC=\left\{TS_{1},TS_{2},\ldots ,TS_{n}\right\}(n>1)$ denote the set of TTSs. The clustering process is as follows. (1) Randomly select an element $TS_{i}\;\left(1\leq i\leq n\right)$ from UC as a cluster seed and search for the elements in UC satisfying the condition $Sim\left(TS_{j},TS_{i}\right)\;\left(j\neq i\right)>simTh$, where simTh is a predefined similarity threshold. (2) Merge the seed TTS as well as the searched TTSs to form a new cluster, which is removed from UC. (3) Steps (1) and (2) are repeated until the set UC is empty. Finally, the TTSs are organized as a set of clusters, $C=\left(c_{1},c_{2},\ldots c_{k}\right)\left(k\geq 1\right)$, where $c_{i}$ (1≤i≤k) denotes a TTS cluster. A sample TTS clustering result is shown in Figure 5, where adjacent TTSs marked with the same color belong to one cluster.

#### 2.3.2. Determining the Coverages of Intersections by Aggregating TTS Clusters

**Figure 6:**
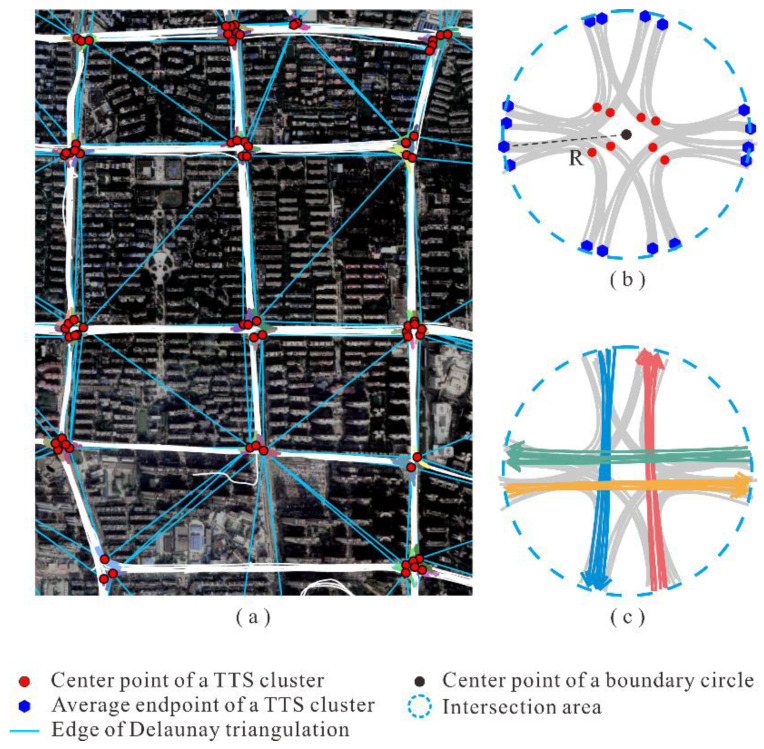
Determining intersection coverages and obtaining clusters of non-turning trajectory segments (non-TTSs): (**a**) applying Delaunay triangulation; (**b**) creating the boundary circle for an intersection; (**c**) obtaining clusters of non-TTSs within an intersection.

Next, the TTS clusters are aggregated to obtain the spatial coverage of the intersections. The procedure is illustrated in Figure 6. Each TTS cluster is represented by its center point, which is the average middle point of all the TTSs in the cluster. As depicted in Figure 6a, a Delaunay triangulation model was built for all the center points to describe the adjacent relationships of the TTS clusters. The triangle edges with lengths exceeding the predefined threshold, disTh, are removed. Subsequently, the TTS clusters whose center points are connected by the remaining triangle edges are merged into a group.

For each TTS cluster group, a circular area is created as the coverage of the intersection. As shown in Figure 6b, the center of the circle is defined as the average coordinates of all center points of the TTS clusters, and the radius is determined as the longest distance between the circle center and all endpoints of the TTS clusters. The endpoints of each TTS cluster are computed by averaging the endpoints of all the TTSs in the cluster. Finally, as shown in Figure 6c, the non-TTSs within the intersection area are extracted by clipping the trajectories with the boundary circle. Note that the obtained non-TTSs are also clustered using the clustering method mentioned earlier.

#### 2.3.3. Generating the Structural Model for Each Intersection

**Figure 7:**
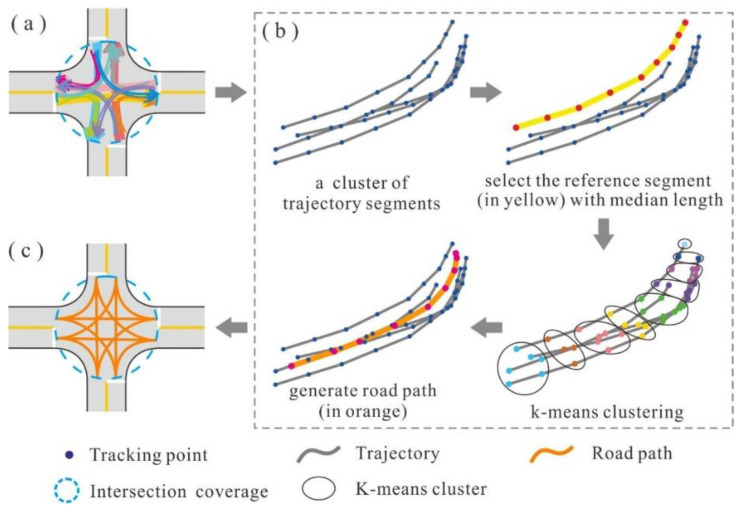
Extracting the internal paths of an intersection: (**a**) TTS and non-TTS clusters (adjacent trajectory segments marked with the same color belong to one cluster); (**b**) road path generation using K-means clustering; (**c**) generated road paths.

The structural model of an intersection can be delineated by extracting the central paths of the associated TTS and non-TTS clusters. The path extraction process is illustrated in Figure 7. For each TTS or non-TTS cluster, the element with the median length is selected as the reference segment. Next, the tracking points of the reference segment are regarded as the initial centers, and the K-means clustering approach is applied to group all tracking points [36,37]. The value of K is set to the number of tracking points in the reference segment. Finally, a path is constructed by connecting the centers of the point groups in chronological order.

## 3. Experiments, Results and Discussion

### 3.1. Experimental Dataset and Pre-Processing Settings

**Figure 8:**
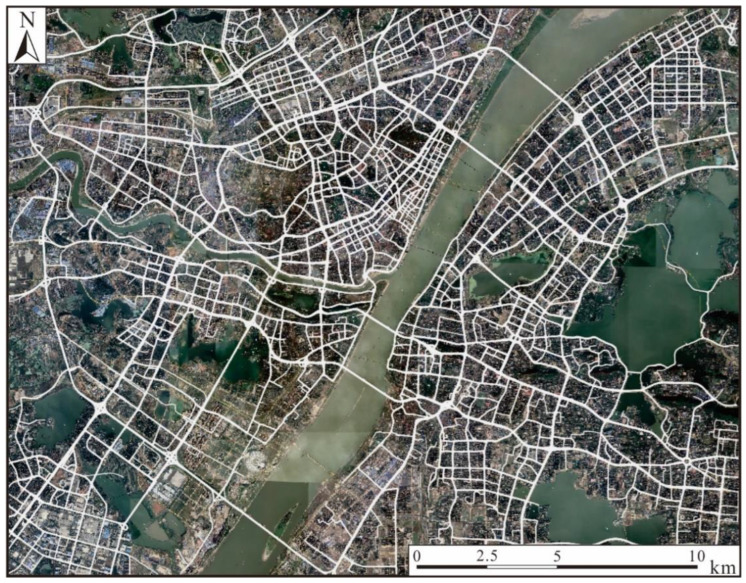
Overview of the vehicle trajectories used for assessing the proposed approach.

To evaluate the effectiveness of the proposed approach, we used a dataset of vehicle trajectories from Wuhan, China. As shown in Figure 8, the dataset covers an area of 25.6 km × 19.9 km. The raw trajectories were captured by GPS trackers in vehicles from online ride-hailing services. During the pre-processing stage, the sampling interval threshold, intTh, was set to 5 s because 97.3% of the tracking points were recorded at intervals of 1 s to 5 s. The maximum valid speed threshold, maxV, was set to 80 km/h, which is the highest speed allowed in the study area. The minLen was set to 200, i.e., a trajectory needs to be composed of at least 200 tracking points, thus providing the LSTM-based model with sequences long enough to learn the implicit features. The interpolation distance threshold, dl, was set to 10 m following the recommendation in the literature [18]. When the Savitzky–Golay filter was implemented, the window size and polynomial order were set to 5 and 1, respectively. As a result, 7492 trajectories were obtained, with a total of 2.9 million tracking points.

### 3.2. Training and Evaluation of the LSTM-Based Model

The LSTM-based model for detecting the TTSs was built on Keras (Available online: https://keras.io/ (accessed on 3 August 2022)), which is an open-source artificial neural network library written in Python with a TensorFlow backend. A total of 308 sample trajectories were selected from the study area to train, validate, and test the model. These trajectories contain TTSs made at various types of intersections, including the T-shaped, Cross-shaped, Y-shaped, and y-shaped intersections, and complex intersections such as interchanges. For each sample trajectory, all line segments were manually labeled as parts of TTSs or non-TTSs based on a human interpretation of the satellite imagery. These sample trajectories were divided into a training set, validation set, and test set, which contain 206, 51, and 51 samples, respectively.

The kappa coefficient [38], an indicator for the consistency tests, was adopted to evaluate the classification performance. ‘Consistency’ indicates whether the prediction results of the trained model are consistent with the actual classification results. This indicator is calculated as follows:

(14) $$Kappa=\frac{2\times \left(n_{TP}\times n_{TN}-n_{FN}\times n_{FP}\right)}{\left(n_{TP}+n_{FP}\right)\times \left(n_{FP}+n_{TN}\right)+\left(n_{TP}+n_{FN}\right)\times \left(n_{FN}+n_{TN}\right)}$$

where $n_{TP}$ is the number of correctly identified line segments belonging to the TTSs, $n_{FP}$ is the number of line segments belonging to the non-TTSs wrongly identified as parts of TTSs, $n_{TN}$ is the number of correctly identified line segments belonging to the non-TTSs, and $n_{FN}$ is the number of line segments belonging to the TTSs wrongly identified as parts of non-TTSs.

To determine the optimal structure of the LSTM-based model, we built several models with different hidden state dimensions in the encoder and decoder layers. Each model was named Model(X), where X represents the dimension of the hidden state (i.e., the number of hidden units in an LSTM cell). All the models were trained using the Adam optimizer with a learning rate of 0.01, and the maximum number of iterations was set to 200. Moreover, each model was implemented with different window sizes to capture the sequence of motion attributes. The constant sequence length within a batch (i.e., *MaxLen*) was set to 500.

**Figure 9:**
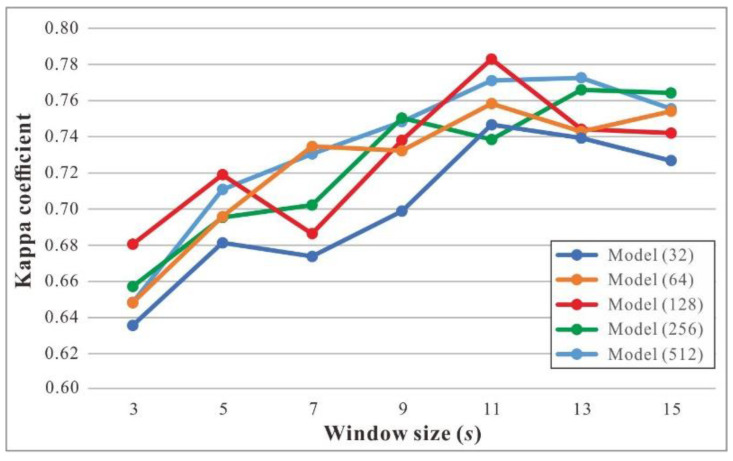
Kappa coefficients achieved by the LSTM-based models with different hidden state dimensions and window sizes on the validation set.

Figure 9 shows the results achieved by models with different dimensions of hidden state and window sizes on the validation set. With an increase in window size, model performance (as determined by the kappa coefficient) increased gradually and then decreased with an increase in window size. When the window size, *s*, was set to 11, four models (i.e., models with hidden state dimensions of 32, 64, 128, and 512) achieved the best performance. In particular, Model (128) achieved the highest kappa coefficient of 0.783. Hence, we chose Model (128) with a window size of 11 as the baseline model for TTS detection. We applied the trained model to the test set, and the kappa coefficient was 0.774, which was close to the performance on the validation set. It proved the effectiveness of the trained model.

Moreover, we conducted an ablation study in which each motion attribute was individually removed. Table 1 lists the results of the ablation test. It was observed that the absence of the turning angle resulted in a significant decline in classification performance, which indicated that the heading change is the most prominent feature for identifying the TTSs. Meanwhile, the use of the other three motion attributes also has a certain positive influence on the improvement in classification performance. These results demonstrate the necessity of the four motion attributes to be considered in the LSTM-based model.

Two popular machine learning methods, i.e., the decision tree (DT) and support vector machine (SVM) [39,40,41,42], were used for comparative testing. The DT-based model was constructed based on Gini impurity and two parameters, i.e., the minimum number of samples required to split an internal node and the minimum number of samples required to be at a leaf node, were set to 2 and 1, respectively. The SVM-based model was constructed on the basis of the radial basis function kernel with its parameter γ computed according to the following equation:

(15) $$\gamma =\frac{1}{n\_features\times var\left(X\right)}\;$$

where n_features denotes the number of features, which was four in this study, and var(X) denotes the variance of the input, i.e., the variance of the four motion attributes.

In addition, two deep learning methods, i.e., the feedforward neural network (FNN) and Transformer, were included for comparison. The Transformer takes advantage of the multi-head self-attention mechanism [43]. It has demonstrated an outstanding sequential processing capability, especially in its ability to deal with long-term dependencies. The hyperparameters for the FNN- and Transformer-based classification models were set based on repeated experiments. The FNN-based model was composed of five fully connected layers, with 128, 64, 16, 8, and 2 units, respectively. As for the Transformer-based model, the number of attention heads was set to four, and eight transformer encoder blocks were stacked.

The results achieved by the different classification models on the test dataset are listed in Table 2. It was observed that the kappa coefficient of the LSTM-based model showed improvements of 0.13–0.14 compared with those of the DT-, SVM-, and FNN-based models, which proves the necessity and effectiveness of sequential modeling. The Transformer took sequential modeling into consideration, which made it superior to the previous models. A closer examination of the results, however, revealed that many of the errors made by the Transformer were around the beginning or end of a TTS. This indicated that the Transformer struggled to determine where a TTS begins or ends. Moreover, situations existed where between two neighboring positive results lay a negative result, leading to an incomplete TTS detected with a gap. Such errors were rare in the results of the LSTM-based model because the prediction result at the previous timestep was directly inputted into the next LSTM cell.

### 3.3. Results of TTS Detection and Intersection Generation

The TTSs were identified by applying the trained LSTM-based model to the entire trajectory dataset; then, the detected TTSs were clustered to obtain the intersection structures. As distance and direction are equally important to cluster the trajectory segments [17], the weights $w_{D}$ and $w_{A}$ in the similarity calculation were set to 0.5. The constant, d, used to normalize the distance measure, was set to 30 m, according to the width of the road surface in the study area. Through repeated experiments, the similarity threshold, simTh, in the TTS clustering process and the threshold, disTh, for aggregating the TTS clusters were set to 0.7 and 80 m, respectively.

**Figure 10:**
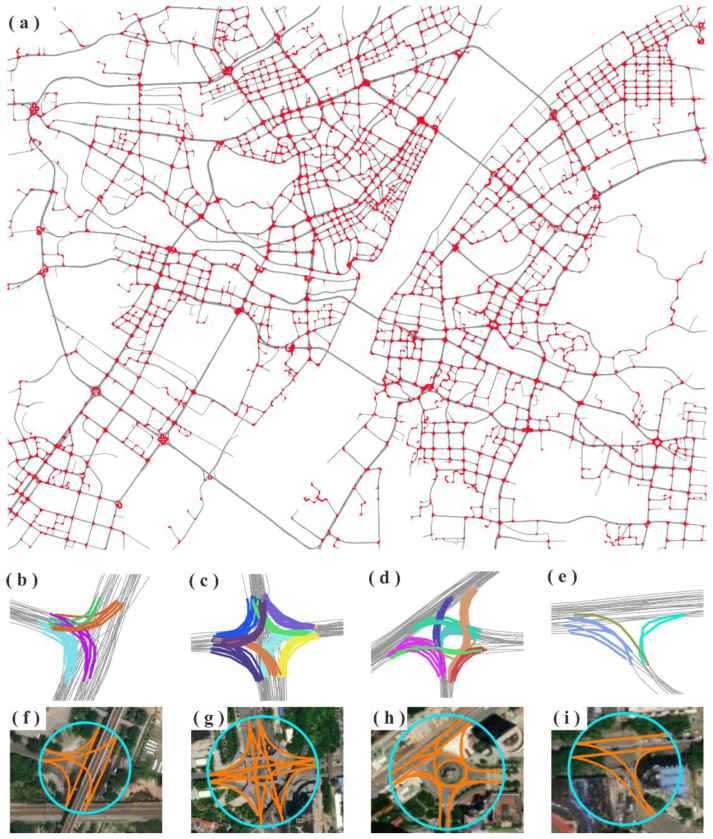
Experimental results: (**a**) overview of the detected TTSs (in red); (**b**–**e**) clustering results for the detected TTSs at intersections with different patterns (adjacent TTSs marked with the same color belong to one cluster); (**f**–**i**) coverages and internal paths generated based on the TTS clusters (circles indicate the boundaries of the detected intersections).

Figure 10a shows the results for the TTS detection over the entire study area. Qualitatively, most of the turns that occurred at the intersections were successfully detected. Figure 10b–i displays close views of the detected TTS clusters and the extracted internal road paths for intersections with typical patterns, indicating that the trained model can accurately detect turns across intersections of different sizes and patterns. Moreover, the coverage and internal paths of the intersections can be well generated based on the clusters of detected TTSs. These positive results may benefit from the ability of the LSTM neural network to capture long-term dependencies in the input sequence, which enables the accurate classification of TTSs and non-TTSs in a trajectory.

For a comparative evaluation, the TTS and intersection detection results are compared with those of the existing approaches. The results from two sub-regions, that is, a central urban region and a semi-urban region, were chosen to evaluate the proposed approach against the existing approaches. The urban region contains a road network with a grid pattern, whereas an irregular road network characterizes the semi-urban region. According to human interpretation based on the high-resolution remote sensing images, there were 86 intersections in the central urban region and 128 intersections in the semi-urban region that were covered by the trajectories.

#### 3.3.1. Comparison of TTS Detection

To evaluate the performance of the LSTM-based TTS detection model, the turning change point pair (TCPP) detection model [17] was implemented as a comparison. Although a TCPP is composed of only two points, it marks the start and end of a TTS, making the result comparable to the result of the proposed method. According to the work of Yang et al. [17], two tracking points, $p_{i}$ and $p_{j}$, in the same trajectory are marked as a TCPP if they satisfy two conditions: (1) the heading difference between $p_{i}$ and $p_{j}$ is greater than 45°, and (2) the time interval between $p_{i}$ and $p_{j}$ is in the range of 8–27 s.

To quantitatively measure the performance of the two methods, the consistency between the detected and manually identified TTSs was analyzed. A consistency ratio (*CR*) metric was defined as follows:

(16) $$CR=\frac{L_{A\cap B}}{L_{A\cup B}}\times \;100\%\;\;\;$$

where $L_{A\cap B}$ and $L_{A\cup B}$ denote the length of the intersection and the length of the union between the detected and manually identified TTSs, respectively.

The *CR* metrics for the TTS detection results that were achieved using the two models are listed in Table 3. It was observed that the *CR* values of the LSTM-based model were higher than those of the TCPP-based model, especially in the semi-urban region, where the road network pattern is less regular. That is because the performance of the TCPP-based model is extremely susceptible to the threshold setting, including the heading change threshold and the time interval threshold. Although those thresholds can be carefully evaluated and selected on a scientific basis by repeated experiments, they still make the TCPP-based method less flexible. For instance, intersections that are far smaller or far larger than an ordinary intersection might need different thresholds for the TCPP-based method to function correctly. On the contrary, the memory cell of LSTM, together with the encoder–decoder architecture, makes the proposed model capable of learning the vehicle’s moving pattern, which is implied in a trajectory before making predictions on a TTS. Therefore, the proposed LSTM-based model has a superior and more robust TTS detection performance.

#### 3.3.2. Comparison of Intersection Generation

The local G* statistic-based approach was implemented for comparison. Different from obtaining intersections by clustering turning segments, this approach detects turning points to generate intersections. According to the recommendations in literature [18], the threshold value for the local G* statistic was set to 2.58, and the minimum number of turning points in each cluster was set to 5. For the quantitative analysis, two evaluation indicators, namely precision and recall, are defined as follows:

(17) $$Precision\;\left(\%\right)=\frac{n_{TP}}{n_{TP}+n_{FP}}\times 100\%\;\;\;$$

(18) $$Recall\;\left(\%\right)=\frac{n_{TP}}{n_{TP}+n_{FN}}\;\times 100\%\;$$

where $n_{TP}$ is the number of correctly detected intersections, that is, the overlap ratio of the intersection boundary circles derived from the proposed method and that the human interpretation is over 80%, $n_{FP}$ is the number of intersections that are either a non-intersection or detected with an incorrect range, i.e., the overlap ratio is less than 80%, and $n_{FN}$ is the number of intersections that were not detected.

**Figure 11:**
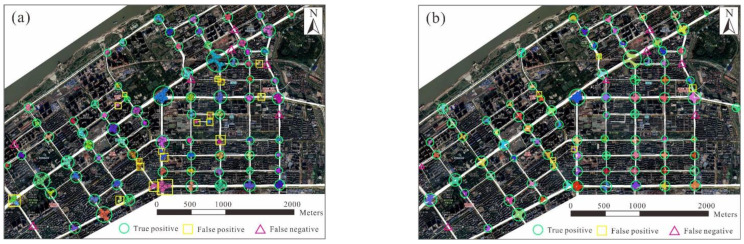
Results of intersection detection in the central urban region: (**a**) using the local G* statistic-based approach and (**b**) using the proposed approach. The points and segments in color are the detected turning points and TTSs, and turning points (TTSs) detected for the same intersection are marked in the same color.

**Figure 12:**
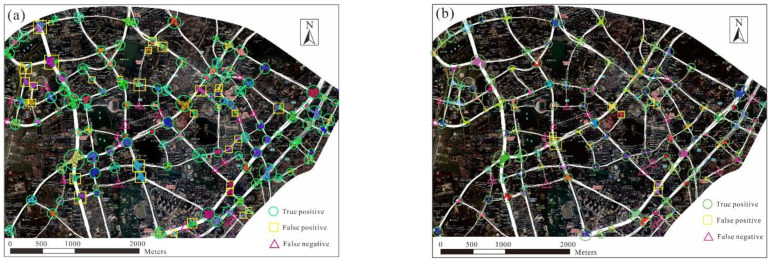
Results of intersection detection in the semi-urban region: (**a**) using the local G* statistic-based approach and (**b**) using the proposed approach. The points and segments in color are the detected turning points and TTSs. Turning points (TTSs) detected for the same intersection are marked in the same color.

Figure 11 and Figure 12 show the results of intersection detection in the central urban and semi-urban regions, respectively. The precision and recall achieved using the two approaches are listed in Table 4. The recall values achieved by the two approaches in the two regions were over 80%, indicating that different types of intersections can be detected based on trajectory data. In comparison, the model performances for the central urban region were much better than those for the semi-urban region. There are two reasons for this difference. First, the trajectory data for the central urban region has a higher coverage of road segments, which is beneficial for the detection of intersections. Second, in the central urban region, the roadways between intersections are approximately straight, which makes it relatively easy to identify the turns occurring at intersections from trajectories.

The proposed approach was superior to the local G* statistic-based approach in accuracy. For the central urban region, the precision values of the proposed and local G* statistic-based approaches were 94.0% and 80.0%, respectively. In particular, 18 intersections detected by the local G* statistic-based approach were inconsistent with the manual identification results, compared with five errors using the proposed approach. Similar findings were obtained for the semi-urban region, where the precision values of the proposed and local G* statistic-based approaches were 94.1% and 73.6%, respectively.

**Figure 13:**
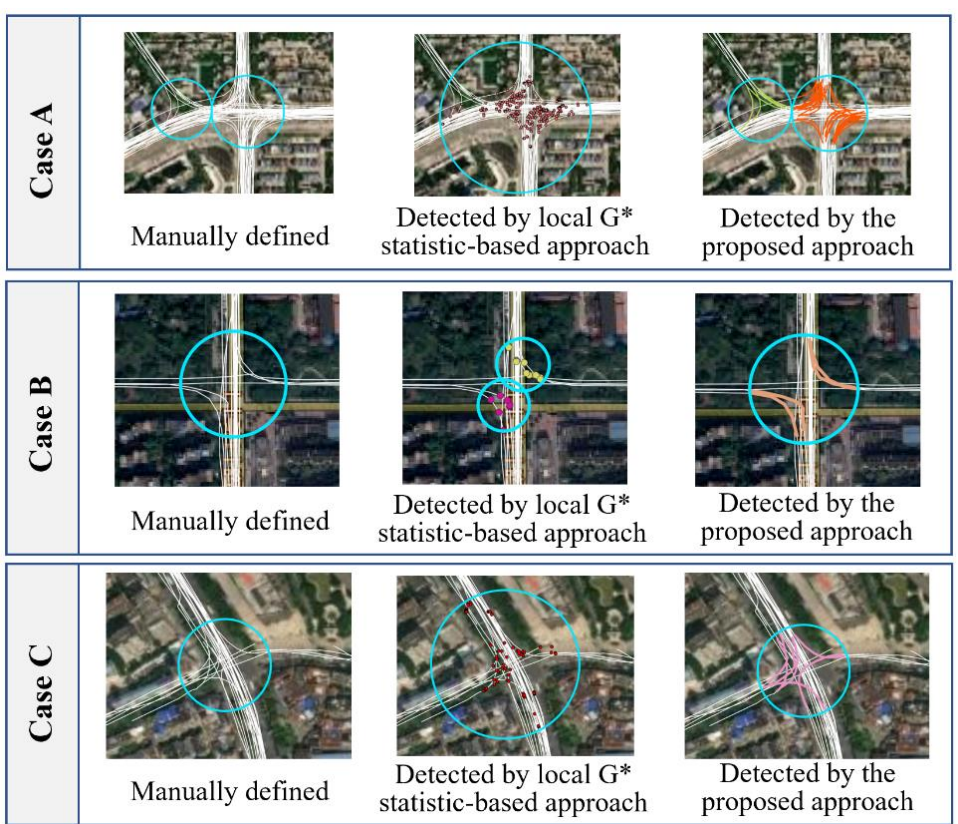
Comparison of the detected intersections using the two approaches. The points and segments in color are the detected turning points and TTSs, and the circles represent the boundaries of the detected intersections.

A comparative analysis revealed that our approach outperforms the local G* statistic-based approach in at least three situations, as illustrated in Figure 13. The first situation occurred in areas with adjacent intersections, where two or more intersections were identified as a single intersection by the local G* statistic-based approach (see Case A). This can be explained by the spatial proximity of intersections, such that the associated turning points were treated as one cluster. Such detection errors were reduced by the newly proposed approach. The second situation occurred in the intersections with a low coverage of trajectories. For example, in Case B, the local G* statistic-based approach treated the associated turning points as two clusters and thus obtained two separate intersections. Third, the coverage of many intersections detected by the local G* statistic-based approach was inaccurate (e.g., Case C). Most of these detection errors were closely related to the misidentification of turning points, and therefore, some tracking points outside of an intersection were added to generate the intersection coverages. Using the proposed approach, this type of incorrect detection was significantly reduced because not only the heading changes but also the speed and acceleration attributes were considered for the decision of turns within the intersection areas. Moreover, the memory capability of LSTM enabled the proposed model to integrate the moving features of the adjacent tracking points, which further improved the ability to detect turns.

## 4. Discussion

The experimental results show that the proposed approach outperforms the local G* statistic-based approach in terms of precision. This improvement benefited from the introduction of the LSTM neural network, which enabled the extraction of high-level moving patterns based on the implicit direction and speed changes in the trajectories. Thus, it helped our approach to better detect the turns (i.e., TTSs) occurring at intersections. Moreover, the difficulty of the parameter settings during the turn detection was alleviated. Existing approaches require the manual determination of appropriate thresholds on heading changes, which can easily lead to incorrect detection due to the diverse structures and sizes of intersections. In contrast, the proposed approach employs the LSTM neural network to learn knowledge to identify the turns from samples, which does not require a manual setting of relevant parameters or rules.

**Figure 14:**
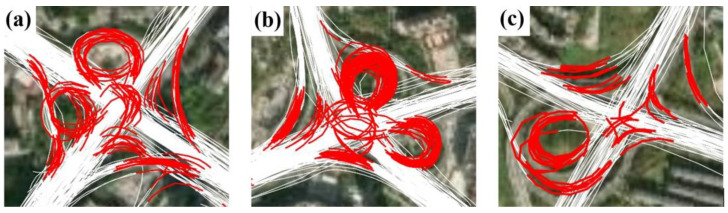
Detection results for TTSs (in red) at three typical complex intersections (**a**–**c**) using the proposed approach.

Despite the relatively good performance of the proposed approach, a small number of intersections were not accurately detected. First, the detection of the y- and Y-shaped intersections needs to be improved. Unlike the T-shaped and cross-shaped intersections, the y- and Y-shaped intersections involve turns with slight heading changes. This makes it difficult to identify the associated TTSs from the trajectories, leading to the missing detection of intersections. Second, it is still challenging to obtain the complete structure of complex intersections. Figure 14 presents a close view of the detected TTSs around several complex intersections. In these examples, many TTSs across intersection areas were missing or not accurately detected, resulting in failures in coverage and internal path generation. The complex intersections, such as interchanges and overpasses, show diverse external shapes and complex internal structures, making their turns much more difficult to characterize. Future work needs to collect more trajectory samples that cover these complex intersections of various sizes and structures and further investigate how those complex intersections can be better tackled.

Moreover, as a supervised learning method, the proposed LSTM-based model depends heavily on high-quality training samples. Labeling sample trajectories requires a certain amount of work and support from professionals. In addition, further investigation is required before using the trained LSTM-based model on other trajectory datasets. For example, applying the model trained on the trajectory samples of Wuhan to detect TTSs in Beijing’s trajectories might see a certain decrease in model performance because of the differences in intersection patterns and local traffic regulations. In such situations, the LSTM-based model may need to be restructured (e.g., the setting of the hidden state in the encode and decode layers) and retrained.

The LSTM-based model alleviates the difficulty of parameter setting and, in turn, detection. However, other steps of the proposed approach, i.e., data pre-processing and intersection structure generation, are still susceptible to the setting of parameters, such as the similarity threshold in TTS clustering and the distance threshold in intersection coverage determination. To solve this problem, an end-to-end approach that generates road intersections from raw trajectories directly using a single neural network or a combination of neural networks needs to be investigated.

## 5. Conclusions

The widespread use of vehicle trajectory data has provided new opportunities for monitoring transportation infrastructures and updating road maps. However, there is still a lack of effective tools for realizing automatic transformation from raw trajectory data to road features. This paper presents a new approach for detecting turns and generating intersections from trajectories. An LSTM-based model was developed to analyze the motion characteristics of the vehicle along each trajectory and to detect TTSs, each of which indicates a turn at an intersection. The detected TTSs were then clustered to obtain the coverage and internal structure of the intersections. The proposed approach was validated using a trajectory dataset obtained from Wuhan. In a central urban region, the intersection detection achieved a precision of 94.0% and a recall of 91.9%. In a semi-urban region, the intersection detection precision and recall were 94.1% and 86.7%, respectively. These results were better than those obtained using the local G* statistic-based approach.

Future work can focus on three aspects to improve the robustness of our approach. First, it is necessary to establish a high-quality sample dataset that contains trajectories covering intersections with various patterns and sizes. Second, other deep learning networks, such as the one-dimension convolutional or graph neural networks, can be considered when searching for more robust models for turn detection. Third, the quality of the generated intersection features, including position accuracy and structural integrity, needs to be fully assessed against official, authoritative road data. Beyond the specific topic of generating road intersections, the proposed approach can potentially be extended to mine the movement behaviors from spatiotemporal trajectory data. This field provides promise for studies of implicit patterns and interpreting human mobility behaviors by spatiotemporal data analytics.

## Figures and Tables

**Table 1:** Ablation study of the four motion attributes in the LSTM-based model.

Tortuosity	Turning Angle	Speed	Acceleration	Kappa Coefficient
√	√	√	√	**0.774**
	√	√	√	0.766
√		√	√	0.614
√	√		√	0.746
√	√	√		0.758

**Table 2:** Comparison of classification performance using different learning methods.

Method	Kappa Coefficient
DT-based model	0.634
SVM-based model	0.644
FNN-based model	0.632
Transformer-based model	0.693
LSTM-based model	**0.774**

**Table 3:** Consistency ratios (CRs) of the TTS detection results using the two approaches.

Study Area	Method	*CR* (%)
Central urban region	TCPP-based model	81.6
	LSTM-based model	92.9
Semi-urban region	TCPP-based model	72.3
	LSTM-based model	88.7

**Table 4:** Statistical summary of the intersection detection results for the central urban and semi-urban regions using the two approaches.

Study Area	Approach	$n_{TP}$	$n_{FP}$	$n_{FN}$	Precision (%)	Recall (%)
Central urban region	Local G* statistic-based approach	72	18	7	80.0	91.1
	Proposed approach	79	5	7	94.0	91.9
Semi-urban region	Local G* statistic-based approach	95	34	18	73.6	84.1
	Proposed approach	111	7	17	94.1	86.7

## Data Availability

The data presented in this study are available on request from the corresponding author. The data are not publicly available due to privacy concerns.
